# Association Between Anatomical Location and Hematoma Expansion in Deep Intracerebral Hemorrhage

**DOI:** 10.3389/fneur.2021.749931

**Published:** 2022-02-02

**Authors:** Lei Song, Xiao-Ming Qiu, Ting-Ting Guo, Hang Zhou, Dong-Fang Tang, Long-Sheng Wang, Yu-Fei Fu, Hui Chen, Hua-Qing Mao, Hai-Bao Wang, Yong-Qiang Yu

**Affiliations:** ^1^Department of Radiology, The First Affiliated Hospital of Anhui Medical University, Hefei, China; ^2^Research Center of Clinical Medical Imaging, Hefei, China; ^3^Anhui Provincial Institute of Translational Medicine, Hefei, China; ^4^Department of Radiology, Huangshi Central Hospital, Affiliated Hospital of Hubei Polytechnic University, Edong Healthcare Group, Huangshi, China; ^5^Department of Radiology, Xiangyang No. 1 People's Hospital, Hubei University of Medicine, Xiangyang, China; ^6^Department of Radiology, Xiangyang Central Hospital, Affiliated Hospital of Hubei University of Arts and Science, Xiangyang, China; ^7^Department of Neurosurgery, Xiangyang Central Hospital, Affiliated Hospital of Hubei University of Arts and Science, Xiangyang, China; ^8^Department of Radiology, The Second Hospital of Anhui Medical University, Hefei, China; ^9^School of Computer Engineering, Hubei University of Arts and Science, Xiangyang, China

**Keywords:** intracerebral hemorrhage, hematoma expansion, deep, location, stroke

## Abstract

**Objective:**

To establish the relationship between hematoma sites of involvement and hematoma expansion (HE) in patients with deep intracerebral hemorrhage (ICH).

**Methods:**

Eligible patients with deep ICH admitted to hospital within 6 hours of onset between 2018 and 2020 were included in this retrospective multi-center study. Individuals with secondary ICH were excluded. The volume of HE was evaluated based on admission and follow-up computed tomography scans. Associations between deep ICH sites of involvement and HE were examined using multivariable logistic regression analysis while adjusting for confounding covariates of HE.

**Results:**

We enrolled 583 individuals from three stroke centers. Data from a final total of 460 patients were used in the analysis; of these patients, 159 (34.6%) had HE. In the crude model without adjustment, external capsule, anterior limb of the internal capsule, and posterior limb of the internal capsule (PLIC) involvement were correlated with HE. After fully adjusted models for sex, age, intraventricular hemorrhage, Glasgow Coma Scale admission score, baseline ICH volume, and time from onset to initial computed tomography, multivariable logistic regression revealed that the PLIC is a robust predictor of HE in patients with deep ICH (adjusted odds ratio = 2.73; 95% confidence interval = 1.75–4.26; *p* < 0.001).

**Conclusion:**

Involvement of the posterior limb of the internal capsule in deep hemorrhage could be a promising predictor of HE.

## Introduction

The Global Burden of Disease 2019 study ranked stroke as the primary cause of disability-adjusted life years in those aged 50 years and older (1). Intracerebral hemorrhage (ICH) is one of the most devastating and fatal subtypes of cerebrovascular accidents, and is associated with mortality rates of up to 40% (2). Approximately 30% of patients with ICH will experience hematoma expansion (HE) within 6 hours after the onset of neurological deficits (3), and HE has long been deemed as an independent predictor for worse outcomes (4). Every 1 mL increase in a hematoma has been estimated to elevate the risk of death by 5% (5). Additionally, attempts to prevent HE are crucial in managing the early phase of ICH, and this is a potential target for hemostasis treatment (6, 7). The timely detection of patients with ICH who have a high risk of hemorrhagic progression is therefore of great importance.

HE is associated with multiple factors, including Glasgow Coma Scale (GCS) score at admission, time from symptom onset to the first computed tomography (CT) scan, hemorrhage location, and initial hematoma volume (8). However, the relationship between hemorrhage location and HE deserves further investigation. Several studies have indicated that patient with deep ICH are more susceptible to HE than those with lobar ICH (9–11). Nevertheless, the inclusion of hemorrhage location may have restricted the applicability of HE differences between lobar and deep groups, which have a different etiology. Spontaneous ICH is overwhelmingly driven by hypertensive angiopathy, especially in deep zones, whereas cerebral amyloid angiopathy is generally cited as the primary reason for ICH at lobar sites (12). However, the heterogeneity between these sites means that there is no sufficient evidence to suggest that HE is more frequently observed in deep ICH than in lobar ICH. Additionally, the basal ganglia-thalamic region has long been viewed as the most frequent site of hemorrhage (7); focusing on deep ICH may help to determine the mechanisms underlying HE.

Thus, additional exploration of the relationship between deep ICH location and HE is warranted. We hypothesized that specific sites of deep ICH affect HE. The current study was performed to test this hypothesis.

## Materials and Methods

### Study Design and Patient Selection

This multi-center, retrospective study was performed at three stroke centers (The First Affiliated Hospital of Anhui Medical University, The Second Hospital of Anhui Medical University, and Xiangyang Central Hospital) from January 2018 to December 2020. Consecutive patients aged >18 years with spontaneous ICH were approached. Eligible individuals were screened in light of the following inclusion criteria: (1) Spontaneous ICH was identified on CT; and (2) baseline CT scans and follow-up CT scans were acquired within 6 h and 48 h after the initial ictus, respectively. The exclusion criteria were as follows: (1) Primary intraventricular hemorrhage (IVH); (2) secondary ICH ascribed to aneurysm, congenital arteriovenous malformation, moyamoya, a tumor-related condition, or hemorrhagic transformation of cerebral infarction; (3) anticoagulant-associated ICH; (4) hematoma evacuation in previous follow-up CT scans; and/or (5) severe imaging artifacts during CT examinations.

### Clinical Data Collection and Image Analysis

Demographic and clinical data were taken from electronic medical records. Data concerning demographics (age and sex), relevant medical conditions (prior ICH, ischemic stroke, hypertension, and diabetes mellitus), and clinical features (alcohol, smoking, international normalized ratio, blood pressure, initial GCS score, and time from symptom onset to first CT scan) were noted. Radiological findings, that is, parenchymal hematoma volume and location, and IVH, were evaluated using baseline and follow-up CT images. Deep ICH was further divided into 6 sites according to a previous study (13), comprising the globus pallidus/putamen (GP/P), anterior limb of the internal capsule (ALIC), external capsule (EC), posterior limb of the internal capsule (PLIC), caudate head, and thalamus. More than one area was reported in the case of hemorrhage that involved more than one site. If minor bleeding was confined to one region, the region were considered to be involved in ICH. For a well-demarcated GP/P, caudate head, and thalamus, implication of more than one-third of the structure was defined as involvement. When ALIC, PLIC, or EC were classified as affected, these regions were considered to be involved. All CT images were randomized and independently reviewed by two highly experienced neuroradiologists (Y-QY and X-MQ) who were blinded to the clinical data. For ambiguous cases or when opinions contradicted, the two neuroradiologists discussed together until they reached a rational conclusion.

Images from CT acquisition with an axial section thickness of 5 mm and no gaps were stored in Digital Imaging and Communications in Medicine format and then imported into the 3D Slicer software (version 4.11.20210226 https://www.slicer.org/). The hematoma was automatically segmented by setting a fixed threshold range (40–100 HU), and the corresponding marked brain tissue or skull outside of the hemorrhage region was manually pruned using Erase tools. Manual layer-by-layer segmentations were performed to connect parenchymal hematomas and IVH hematomas. Finally, the hemorrhage volume was calculated by subtracting the initial CT from the follow-up CT. The criteria for HE were determined by referring to prior studies (14, 15), which included the following four types: parenchymal hematomas ≥6 mL or >33% increase according to CT scans at admission and during follow-up; IVH expansion ≥1 mL on comparison of follow-up and baseline CT scans; any new IVH expansions on follow-up CT scans.

### Statistical Analyses

The Kolmogorov–Smirnov test was applied to test the normality of data distribution. Normally distributed variables are expressed as the mean ± standard deviation (SD), and non-normally distributed variables are expressed as the median ± interquartile range. Categorical data are presented as percentages (%). Between-group differences were analyzed using Student's *t*-tests (or Mann–Whitney *U*-tests) for continuous variables, and Chi-square (χ^2^) tests (or Fisher's exact tests) for categorical variables, as appropriate.

The relevant risk factors for HE were identified using univariate analysis, and the odds ratio (OR) and confidence intervals (95% CIs) were calculated. The association between deep hematoma sites and HE were estimated using multivariable binary logistic regression models after adjusting for potential confounders, such as age, sex, time from symptom onset to initial CT scan, baseline hematoma volume, admission GCS score, and IVH, as informed by prior studies. We further explored the relationship between specific regions of deep hemorrhages and HE using stratification analysis. The standard level of statistical significance was determined at *p* < 0.05. All statistical analyses were performed using R software (version 4.0.3).

## Results

### Subject Population and Characteristics

This retrospective study enrolled 583 individuals from three stroke centers. A total of 460 patients with deep ICH were enrolled in the analytic sample, of whom 159 (34.6%) had HE. We removed 123 patients for several reasons, as follows: infratentorial ICH (*n* = 44), lobar ICH (*n* = 70), and combined deep and lobar sites (*n* = 9). The flowchart of participant selection is presented in Supplementary Figure 1.

Differences in baseline characteristics of deep ICH were notable between groups with and without HE. Patients with HE had worse GCS scores, had a shorter time from onset to CT, and had a larger baseline ICH volume than patients without HE (*p* < 0.05). No clinically relevant disparities in demographic characteristics or medical history were noted. Similarly, there were no remarkable between-group differences in the international normalized ratio, intraventricular hemorrhage, systolic blood pressure, or diastolic blood pressure (Table 1).

**Table 1:** Baseline characteristics of deep ICH.

**Variables**	**HE –ve**	**HE +ve**	***P*-value**
	**(*N* = 301)**	**(*N* = 159)**	
**Demographic**			
Age, years, mean (SD)	60.4 (12.2)	59.7 (13.1)	0.526
Sex, male, n (%)	200 (66.4)	109 (68.6)	0.647
**Medical history**, ***n*** **(%)**			
Prior ICH	26 (8.6)	14 (8.8)	0.952
Ischemic stroke	26 (8.6)	23 (14.5)	0.054
Hypertension	214 (71.1)	111 (69.8)	0.773
Diabetes mellitus	23 (7.6)	20 (12.6)	0.084
**Clinical features**			
Alcohol, *n* (%)	93 (30.9)	53 (33.3)	0.593
Smoking, *n* (%)	93 (30.9)	49 (30.8)	0.986
INR, median (IQR)	1.0 [0.9–1.0]	1.0 [0.9–1.0]	0.406
SBP, mmHg, mean (SD)	184.4 (29.8)	186.8 (31.1)	0.421
DBP, mmHg, mean (SD)	99.9 (17.4)	101.5 (16.7)	0.366
Admission GCS, median (IQR)	12 [10–14]	11 [7–13]	<0.001
Time from onset to CT, hours, median (IQR)	2.4 [1.5–3.7]	1.9 [1.2–3.0]	0.024
**Radiographic features**			
Baseline ICH volume, mL, median (IQR)	12.3 [6.6–20.5]	16.7 [8.6–30.1]	<0.001
IVH, n (%)	97 (32.2)	58 (36.5)	0.359
**Involved locations**, ***n*** **(%)**			
GP/P	211 (70.1)	116 (73.0)	0.520
EC	112 (37.2)	86 (54.1)	<0.001
Caudate head	9 (3.0)	11 (6.9)	0.049
ALIC	25 (8.3)	28 (17.6)	0.003
PLIC	128 (42.5)	109 (68.6)	<0.001
Thalamus	103 (34.2)	53 (33.3)	0.849

*HE, hematoma expansion; –ve, negative; +ve, positive; SBP, systolic blood pressure; DBP, diastolic blood pressure; GCS, Glasgow Coma Scale; CT, computed tomography; IVH, intraventricular hemorrhage; INR, international normalized ratio; ICH, intracerebral hemorrhage; GP/P, globus pallidus/putamen; EC, external capsule; ALIC, anterior limb of the internal capsule; PLIC, posterior limb of the internal capsule; SD, standard deviation; IQR, interquartile range*.

**Figure 1:**
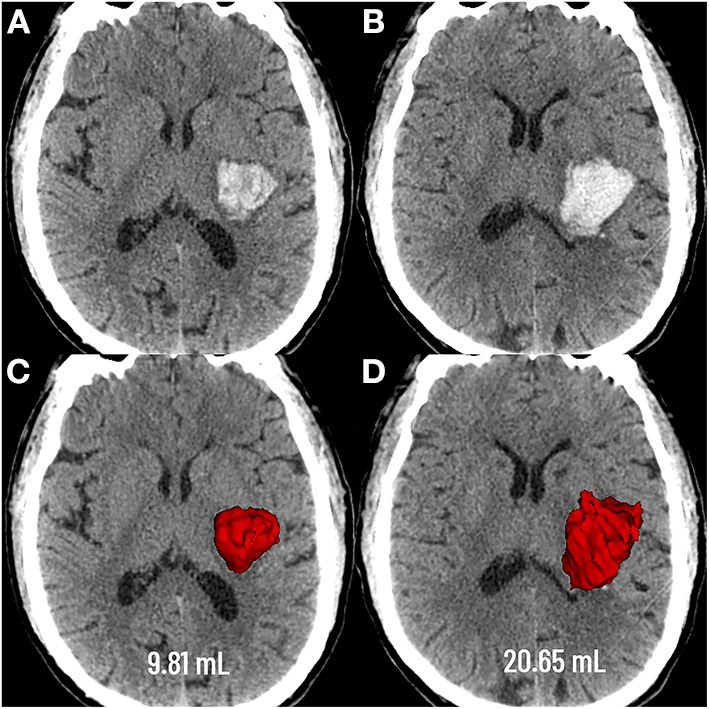
Graphic representation of the extent of deep hematoma involvement on non-contrast CT. Deep hematoma involving the PLIC was seen as early as 3.4 h following neurological deficits **(A)**, with progressive expansion over the subsequent 23.1 h **(B)**. Baseline CT showed a hematoma volume of 9.81 mL **(C)**, whereas the volume was 20.65 mL at the follow-up CT **(D)**. PLIC, posterior limb of the internal capsule.

The involved anatomic sites in deep ICH included 15 single or multiple combinations. The most frequent patterns were involvement of the PLIC and thalamus (*n* = 90), GP/P alone (*n* = 86), GP/P, EC, and PLIC (*n* = 82), and GP/P and EC (*n* = 63). Details of the most frequent patterns of deep ICH are shown in Supplementary Table 1. The analysis of deep ICH locations revealed that involvement of the EC, ALIC, and PLIC was more prevalent in the group with HE (*p* < 0.05), whereas involvement of the other sites did not differ between groups (Figure 1).

### Association Between Involved Locations and HE

In the crude model without adjustment, EC, ALIC, and PLIC involvement were correlated with HE [*OR* = 1.99 (95% CIs = 1.35–2.94); *OR* = 2.36 (95% CIs = 1.32–4.21), and *OR* = 2.95 (95% CIs = 1.96–4.42), respectively]. After minimal adjustments for sex and age, multivariable logistic regression revealed that the EC (*OR* = 1.98; and 95% *CIs* = 1.34–2.94; *p* < 0.001), ALIC (*OR* = 2.37; 95% *CIs* = 1.33–4.23; *p* = 0.003), and PLIC (*OR* = 3.00; 95% *CIs* = 1.99–4.51; *p* < 0.001) were associated with HE. In fully adjusted models for sex, age, IVH, admission GCS score, baseline ICH volume, and time from onset to CT, the EC and ALIC were not predictive of HE, whereas the PLIC (*OR* = 2.73; 95% *CIs* = 1.75–4.26; *p* < 0.001) remained predictive of HE (Table 2).

**Table 2:** Crude and adjusted multivariable logistic regression analysis for the association between deep ICH sites of involvement and hematoma expansion.

**Variables**	**No**.	**Crude model**	***p-*value**	**Minimally adjusted model**	***p-*value**	**Fully adjusted model**	***p-*value**
		**OR (95% CI)**		**OR (95% CI)**		**OR (95% CI)**	
GP/P	327	1.15 (0.75, 1.77)	0.521	1.13 (0.73, 1.74)	0.596	0.54 (0.29, 0.99)	0.047
EC	198	1.99 (1.35, 2.94)	<0.001	1.98 (1.34, 2.94)	<0.001	1.31 (0.77, 2.22)	0.316
Caudate head	20	2.41 (0.98, 5.95)	0.056	2.50 (1.01, 6.18)	0.048	1.24 (0.44, 3.44)	0.685
ALIC	53	2.36 (1.32, 4.21)	0.004	2.37 (1.33, 4.23)	0.003	1.24 (0.63, 2.45)	0.530
PLIC	237	2.95 (1.96, 4.42)	<0.001	3.00 (1.99, 4.51)	<0.001	2.73 (1.75, 4.26)	<0.001
Thalamus	156	0.96 (0.64, 1.44)	0.849	0.99 (0.65, 1.49)	0.946	1.76 (0.98, 3.17)	0.060

*Crude model: Without adjustment*.

*Minimally adjusted model: Covariates were adjusted for age, sex*.

*Fully adjusted model: Covariates were adjusted for age, sex, IVH, admission GCS score, Baseline ICH volume, and time from onset to CT*.

*OR, odds ratio; CI, confidence interval; GP/P, globus pallidus/putamen; EC, external capsule; ALIC, anterior limb of the internal capsule; PLIC, posterior limb of the internal capsule*.

### Subgroup Analyses

**Figure 2:**
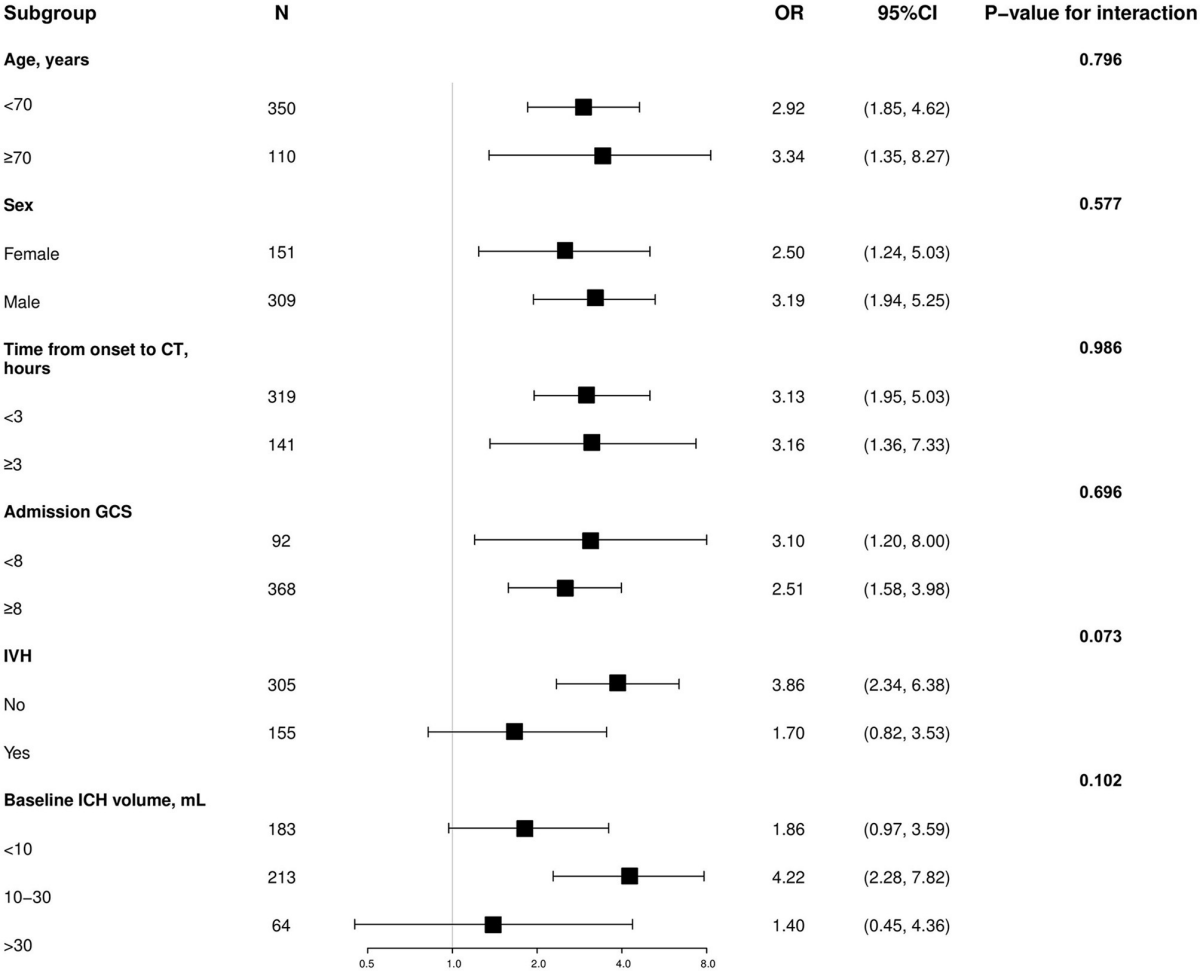
Subgroup analysis of the association between hematoma expansion and the PLIC. OR, odds ratio; CI, confidence interval; GCS, Glasgow Coma Scale; IVH, intraventricular hemorrhage; CT, computed tomography; ICH, intracerebral hemorrhage.

To elucidate the suitability and applicability of the PLIC predicting HE among different potential clinical confounders, we conducted subgroup analyses. The impact of the PLIC on HE was not affected by age, sex, time from onset to CT, admission GCS score, IVH, or baseline ICH volume (value of p for interaction = 0.073–0.986; Figure 2).

## Discussion

In this relatively large retrospective multi-center study, we demonstrated a strong relationship between anatomic zones and HE in patients with deep ICH. Previously identified vital factors related to HE are larger baseline hematoma volumes, shorter time from onset to initial CT, lower GCS score at admission, and IVH (3, 16, 17). When controlling for these potential confounders in the multivariable logistic regression, we found that any deep ICH that affects the PLIC is still a vigorous driver of HE alone. Similarly, subgroup analyses including relevant variables revealed a similar result.

Many previous studies have focused on the occurrence of hematoma growth, with an emphasis on the ability to predict HE (3, 18, 19). That is to say, differences between deep and lobar hemorrhages, which may affect HE, were not taken into consideration. Deep hemorrhages were found to be overwhelmingly driven by hypertension, whereas cerebral amyloid angiopathy has been commonly cited as the primary reason for lobar locations, although hypertension was also a pivotal cause (12). In most previous studies (9–11), the implications of hematoma heterogeneity have typically been disregarded, which means that there has been no sufficient evidence to suggest that HE is more frequently observed in deep than in lobar groups. Therefore, we did not include patients with lobar hemorrhages in the present study.

It is increasingly urgent and imperative to identify HE, as growing evidence has revealed that deep sites contribute to worse clinical outcomes after ICH (7, 9, 11). The influence of deep HE on the clinical severity is dependent on the functional integrity of impaired fiber bundles and the specific anatomic regions of lesions. Damage to pyramidal tracts caused by HE of the internal capsule not only triggers physical disability but also gives rise to subjective discomfort and distress (20, 21). Once thalamic ICH occurs, there is a higher probability that blood will leak into the ventricles and that massive bleeding will add pressure to the brainstem, resulting in acute obstructive hydrocephalus and even rapid death (22). However, previous investigations into the latent association between deep locations and HE have been limited. Consequently, identification of the risk of HE based on the involvement of specific areas is critical for stratifying patients for therapeutic interventions.

The precise mechanism underlying the correlation between PLIC involvement and HE, however, remains unclear. It possible that specific anatomic characteristics provide the foundation for hemorrhagic progression. Since projection fibers are primarily located in the internal capsule, and are most abundant in the PLIC (23), blood to the PLIC region is supplied by branches from multiple arteries (24). One large study has also indicated that hematoma involving the PLIC is strongly correlated with a worse prognosis (13), and that HE itself is a critical predictor of ICH outcome (3), which underlines the potential link between the PLIC and HE. An additional possible explanation for our novel finding is that the comparatively larger contact area between the PLIC and lateral ventricle increases the risk of HE. Hematoma involving the PLIC might result in higher pressures, with effects spreading to the lateral ventricle. Deng et al. (25) also proposed that hematoma ventricle distance can predict poor outcomes in ICH, which is well-aligned with the current findings. It is puzzling that the thalamus, which is closer to the lateral ventricles, should theoretically be more prone to HE, but we found no evidence for this. Neisewander et al. (26) divided thalamic hemorrhage into six parts, and they found that posterior and lateral bleeds were less likely to cause neurological deterioration than global bleeds. However, our study is only a crude analysis of the involvement of thalamic hemorrhage, which may have obscured the natural conditions. As such, future studies focusing on the influence of specific regions of the thalamus on hematoma growth are warranted.

Our study has several strengths. We used data from three centers, which better exemplifies the real-world heterogeneity, with much more diverse and representative cases of ICH than single-center studies. Another advantage of our research lies in the accurate measurement of ICH volumes based on three-dimensional analysis, which reduces measurement errors caused by irregular hematoma and intraventricular hemorrhage.

However, the current investigation has some limitations. First, The patients were recruited retrospectively from the existing database, and so selection bias cannot be ruled out. Second, it is difficult to estimate the precise time of onset since neurological symptoms are found suddenly by eyewitness statements. If the actual onset was not witnessed, the time of onset of ICH symptoms was defined as the moment at which the patient was last seen to be normal, which may have resulted in recall bias. Finally, follow-up CT scans for a small portion of patients are determined by the actual clinical situation, which may underestimate the detection rate of real HE.

## Conclusions

For patient with deep ICH, we suggest that more attention be paid to the hematoma location, because there is a strong association between hemorrhage involving the posterior limb of the internal capsule and HE.

## Data Availability Statement

The datasets presented in this article are not readily available because requests to access them must first be approved by the stroke centers that provided them. Requests to access the datasets should be directed to Yong-Qiang Yu, cjr.yuyongqiang@vip.163.com.

## Ethics Statement

The Ethical Review Committees of Xiangyang Central Hospital, the First Affiliated Hospital of Anhui Medical University, and the Second Hospital of Anhui Medical University reviewed and approved this study. The data from three centers were anonymous and processed in a confidential manner. The request for written informed consent was waived due to the retrospective design of the present study. Written informed consent was obtained from the individual(s) for the publication of any potentially identifiable images or data included in this article.

## Author Contributions

LS and Y-QY involved in the study design, interpretation of data, and study supervision. LS contributed to data collection, data analysis, data management, and manuscript writing. T-TG contributed to the study design, manuscript review, and proofreading. X-MQ contributed to the data analysis and proofreading. HZ, L-SW, D-FT, HC, and H-BW responsible for data collection. Y-FF contributed to data analysis. H-QM involved in the data analysis and grammar. All authors contributed to the article and approved the submitted version.

## Funding

This study was supported by grants from the National Natural Science Foundation of China, Grant/Award Numbers: 81771817, 81801679, and 82071905.

## Conflict of Interest

The authors declare that the research was conducted in the absence of any commercial or financial relationships that could be construed as a potential conflict of interest.

## Publisher's Note

All claims expressed in this article are solely those of the authors and do not necessarily represent those of their affiliated organizations, or those of the publisher, the editors and the reviewers. Any product that may be evaluated in this article, or claim that may be made by its manufacturer, is not guaranteed or endorsed by the publisher.
