# Supplementary material for: Association Between Anatomical Location and Hematoma Expansion in Deep Intracerebral Hemorrhage
Source: Front Neurol. 2022 Feb 2;12:749931. doi: 10.3389/fneur.2021.749931 (PMC8847973; doi:10.3389/fneur.2021.749931)
Supplement: Supplementary file 1 [file Table_1.DOCX]

Supplementary Material


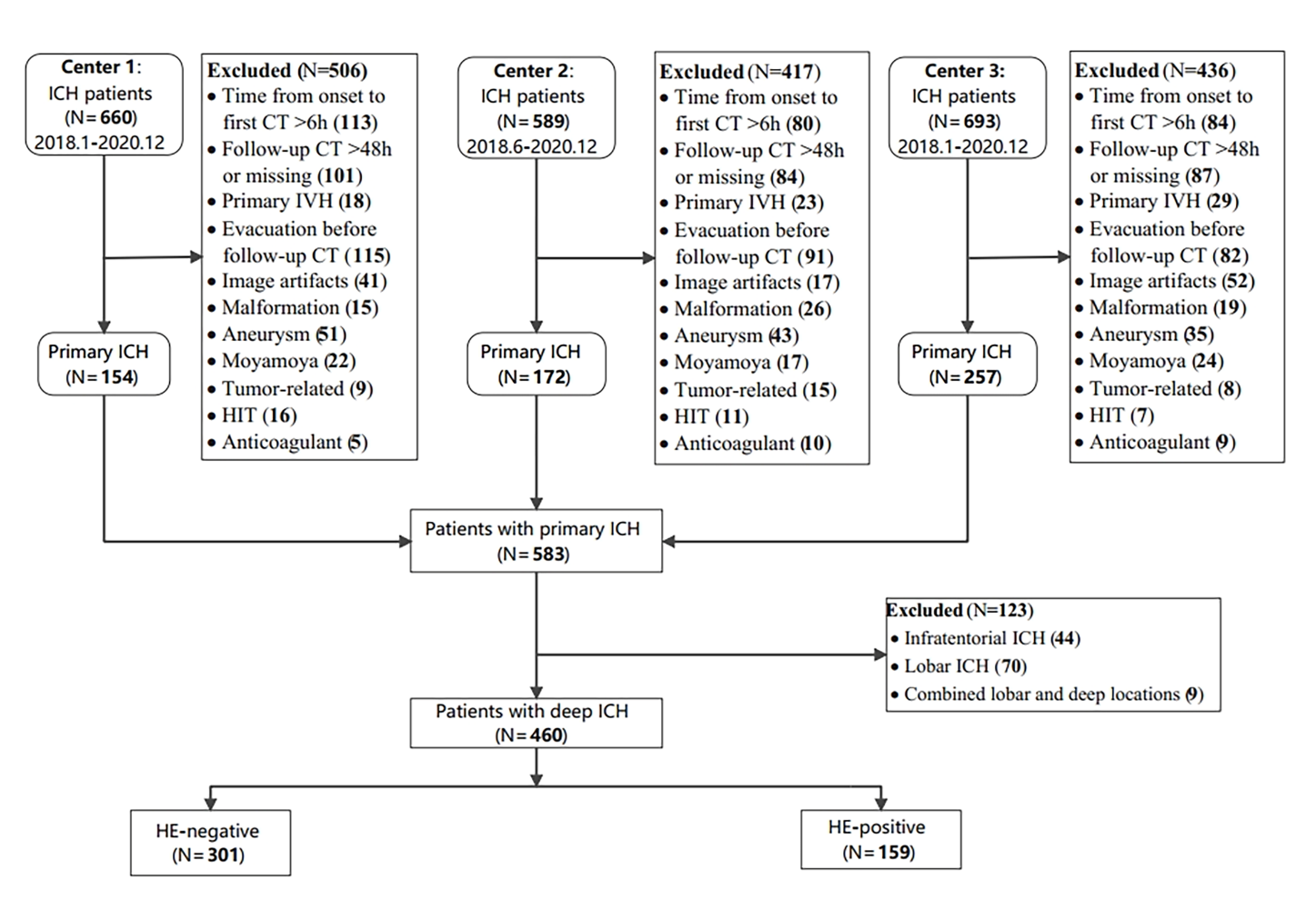


**Figure S1** | Cohort selection flowchart

**Table S1**. Frequent patterns of deep ICH

| **Number of patients** | **Deep ICH locations** |
| --- | --- |
| 90 | Thalamus and posterior limb of the internal capsule |
| 86 | Globus pallidus/putamen |
| 82 | Globus pallidus/putamen, external capsule and posterior limb of the internal capsule |
| 63 | Globus pallidus/putamen and external capsule |
| 41 | Thalamus |
| 27 | Globus pallidus/putamen and posterior limb of the internal capsule |
| 13 | Globus pallidus/putamen, external capsule and anterior limb of the internal capsule |
| 13 | Globus pallidus/putamen, external capsule, posterior limb of the internal capsule and anterior limb of the internal capsule |
| 12 | Thalamus, posterior limb of the internal capsule and globus pallidus/putamen |
| 11 | Thalamus, posterior limb of the internal capsule, globus pallidus/putamen and external capsule |
| 9 | Globus pallidus/putamen, external capsule, anterior limb of the internal capsule and caudate |
| 5 | Globus pallidus/putamen, external capsule, posterior limb of the internal capsule, anterior limb of the internal capsule and caudate |
| 4 | Globus pallidus/putamen, posterior limb of the internal capsule and anterior limb of the internal capsule |
| 2 | Thalamus, posterior limb of the internal capsule, globus pallidus/putamen, external capsule and anterior limb of the internal capsule |
| 2 | Anterior limb of the internal capsule and caudate |
